# Supplementary material for: The Effects of Irrigation, Genotype and Additives on Tef Silage Making
Source: Animals (Basel). 2023 Jan 29;13(3):470. doi: 10.3390/ani13030470 (PMC9913197; doi:10.3390/ani13030470)
Supplement: Supplementary file 1 [file animals-13-00470-s001.zip › animals-2059177-supplementary.pdf]

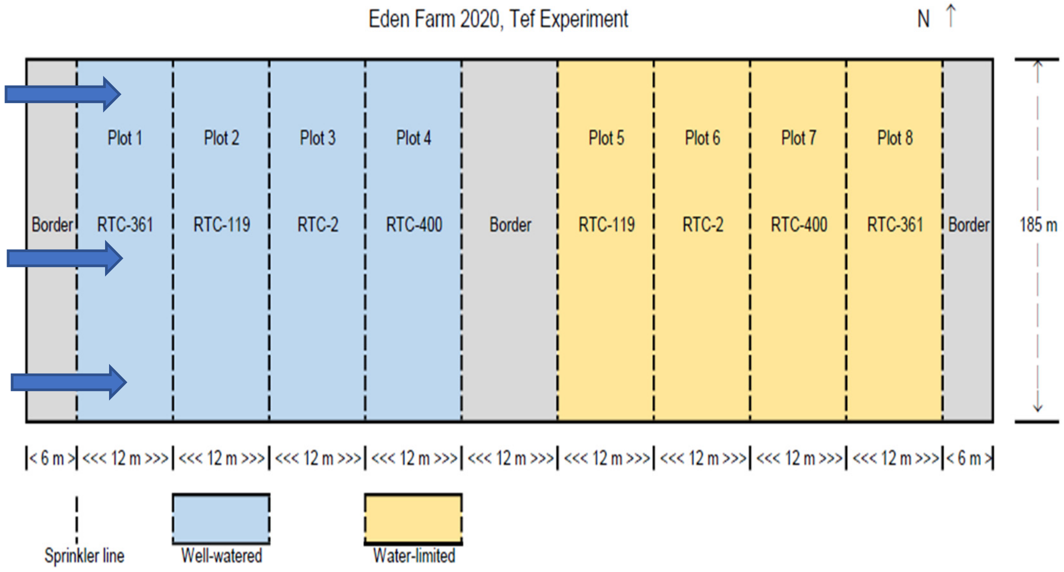

Figure S1: Field experimental design showing the different irrigation regimes; Well-watered (100% irrigation; light blue) and Water-limited (75% irrigation; yellow). Note: Each plot was sampled samples at 3 different location as indicated by the blue arrows.

Table S1. The effect of irrigation, genotype and additives on tef silage parameters at day 0 and 5 days after aerobic exposure.

The effect of irrigation, genotype and additives on pH of tef silage at day 0 and 5 days after aerobic exposure

pH at day 0

	Water-limited (75%)					Well-watered (100%)					Effect (*P*-value)						
**Genotype**	**CTL[1]**	**HI[2]**	**MOL[3]**	**MOL+HI[4]**	**SEM**	**CTL**	**HI**	**MOL**	**MOL+HI**	**SEM**	**Irr[5]**	**Gen[6]**	**Add[7]**	**IrrxGen[8]**	**IrrxAdd[9]**	**GenxAdd[10]**	**IrrxGenxAdd[11]**
RTC-2	4.3*	4.2*	4.1*	4.2*	0.03	5.9^{a}	6.0^{a}	6.1^{a}	5.0^{b}	0.14	<.0001	<.0001	0.0004	<.0001	0.0005	0.0014	<.0001
RTC-119	4.3	4.6	4.5	4.5	0.05	4.6	4.4	4.4	4.3	0.05							
RTC-361	4.2*	4.2*	4.1	4.0*	0.02	4.8	4.7	4.5	4.8	0.05							
RTC-400	4.1*	4.1	4.0	4.1	0.02	4.7	4.5	4.4	4.4	0.04							

pH at day 5

	Water-limited (75%)					Well-watered (100%)					Effect (*P*-value)						
**Genotype**	**CTL[1]**	**HI[2]**	**MOL[3]**	**MOL+HI[4]**	**SEM**	**CTL**	**HI**	**MOL**	**MOL+HI**	**SEM**	**Irr[5]**	**Gen[6]**	**Add[7]**	**IrrxGen[8]**	**IrrxAdd[9]**	**GenxAdd[10]**	**IrrxGenxAdd[11]**
RTC-2	4.3*	4.2*	4.0*	4.1*	0.05	5.6^{a}	5.0^{a}	5.7^{a}	4.9^{b}	0.11	<.0001	<.0001	0.004	<.0001	0.0038	0.0017	0.0015
RTC-119	4.3	4.5	4.4	4.4	0.04	4.5	4.3	4.7	4.2	0.10							
RTC-361	4.1*	4.0*	4.0*	3.9*	0.02	4.8	4.8	4.7	4.9	0.04							
RTC-400	4.0	4.0	3.9	4.1	0.03	4.4	4.5	4.3	4.3	0.02							

The effect of irrigation, genotype and additives on IVDMD of tef silage at day 0 and 5 days after aerobic exposure

IVDMD (%) at day 0

	Water-limited (75%)					Well-watered (100%)					Effect (*P*-value)						
**Genotype**	**CTL[1]**	**HI[2]**	**MOL[3]**	**MOL+HI[4]**	**SEM**	**CTL**	**HI**	**MOL**	**MOL+HI**	**SEM**	**Irr[5]**	**Gen[6]**	**Add[7]**	**IrrxGen[8]**	**IrrxAdd[9]**	**GenxAdd[10]**	**IrrxGenxAdd[11]**
RTC-2	68.9^{ab}	71.7^{a}*	57.4^{b}	72.1^{a}*	2.3	58.0	54.8	57.3	54.3	0.94	<.0001	<.0001	0.0231	<.0001	0.1062	<.0001	0.0004
RTC-119	58.5	50.0	60.7	62.2	1.8	55.2	53.6	54.3	66.7	1.64							
RTC-361	61.9	57.7	66.1	67.9*	1.5	57.5	57.0	60.1	53.9	0.80							
RTC-400	61.5	62.0	69.8	62.4	1.1	55.2^{b}	70.4^{a}	68.8^{a}	62.6^{ab}	2.01							

IVDMD (%) at day 5

	Water-limited (75%)					Well-watered (100%)					Effect (*P*-value)						
**Genotype**	**CTL[1]**	**HI[2]**	**MOL[3]**	**MOL+HI[4]**	**SEM**	**CTL**	**HI**	**MOL**	**MOL+HI**	**SEM**	**Irr[5]**	**Gen[6]**	**Add[7]**	**IrrxGen[8]**	**IrrxAdd[9]**	**GenxAdd[10]**	**IrrxGenxAdd[11]**
RTC-2	74.3*	75.4*	66.5*	72.9*	2.1	54.9	43.4	48.8	48.0	1.46	<.0001	<.0001	0.0323	<.0001	0.7758	0.5249	0.0008
RTC-119	52.9	47.1	61.2	52.3	2.0	53.5	50.6	50.3	62.0	1.84							

RTC-361	58.1	53.0	55.3	61.1*	1.6	45.2	42.5	43.7	42.4	0.86
RTC-400	64.3	51.2	52.5	57.7	1.7	58.8	64.1	64.737	59.7	1.59

The effect of irrigation, genotype and additives on lactic acid bacteria count of tef silage at day 0

LAB (log_{10} CFU g/DM)

	Water-limited (75%)					Well-watered (100%)					Effect (*P*-value)						
**Genotype**	CTL[1]	HI[2]	MOL[3]	MOL+HI[4]	SEM	CTL	HI	MOL	MOL+HI	SEM	Irr[5]	Gen[6]	Add[7]	IrrxGen[8]	IrrxAdd[9]	GenxAdd[10]	IrrxGenxAdd[11]
RTC-2	9.8	9.8	9.7	9.8	0.03	10.7	10.6	10.8	11.0	0.08	<.0001	<.0001	0.0109	<.0001	0.0044	<.0001	0.0002
RTC-119	9.9	9.3	9.9	9.8	0.11	10.5	10.3	10.5	10.5	0.09							
RTC-361	9.6[a]	10.0[a]	N.D[b]	4.0[b]	1.74	10.8	10.9	10.8	10.8	0.02							
RTC-400	9.3	9.8	9.7	10.0	0.10	9.8	9.9	10.1	10.1	0.04							

The effect of additives on the VFA profile of tef silage at day 0 and 5 days after aerobic exposure

VFA (g/100g DM) at day 0

	Additive					Effect (*P*-value)						
**VFA**	CTL[1]	HI[2]	MOL[3]	MOL+HI[4]	SEM	Irr[5]	Gen[6]	Add[7]	IrrxGen[8]	IrrxAdd[9]	GenxAdd[10]	IrrxGenxAdd[11]
Ethanol	0.02	0.02	0.02	0.04	0.004	0.1090	0.5888	0.2772	0.0109	0.5860	0.9206	0.9366
Acetic acid	0.51	0.90	0.69	0.87	0.057	0.4397	0.2055	0.0448	0.0136	0.8742	0.3897	0.4265
Propionic acid	N.D	0.01	0.03	0.15	0.028	0.1548	0.0310	0.2195	0.1055	0.2426	0.1085	0.1277
Butyric acid	0.01	N.D	0.01	N.D	0.002	0.1259	0.0839	0.1079	0.0019	0.1715	0.0398	0.0587
Total	0.54[b]	0.94[ab]	0.76[ab]	1.06[a]	0.060	0.9005	0.9420	0.0229	0.0966	0.5389	0.6255	0.7418

VFA (g/100g DM) at day 5

	Additive											
**VFA**	CTL[1]	HI[2]	MOL[3]	MOL+HI[4]	SEM	Irr[5]	Gen[6]	Add[7]	IrrxGen[8]	IrrxAdd[9]	GenxAdd[10]	IrrxGenxAdd[11]
Ethanol	N.D	N.D	N.D	N.D	0.000	0.5844	0.8346	0.8148	0.8346	0.8148	0.9646	0.9646
Acetic acid	0.32	0.39	0.48	0.33	0.048	0.0517	0.0704	0.56	0.0328	0.2638	0.5222	0.3212
Propionic acid	0.20[a]	N.D[b]	0.12[ab]	N.D[b]	0.036	0.0006	0.0034	0.0033	0.0034	0.0033	<.0001	<.0001
Butyric acid	0.02	0.16	0.03	0.13	0.024	0.1721	0.2130	0.0918	0.7556	0.1026	0.4043	0.068
Total	0.55	0.54	0.62	0.46	0.055	0.6078	0.2312	0.729	0.1647	0.5983	0.0403	0.0056

The effect of irrigation, genotype and additives on lactic acid concentration of tef silage at day 0 and 5 days after aerobic exposure

Lactic acid concentration (g/100g DM) at day 0

	Water-limited (75%)					Well-watered (100%)					Effect (*P*-value)						
**Genotype**	CTL[1]	HI[2]	MOL[3]	MOL+HI[4]	SEM	CTL	HI	MOL	MOL+HI	SEM	Irr[5]	Gen[6]	Add[7]	IrrxGen[8]	IrrxAdd[9]	GenxAdd[10]	IrrxGenxAdd[11]
RTC-2	0.2	0.2	0.2	0.3	0.04	0.7	0.2	0.1	0.2	0.11	0.1711	0.0001	0.1986	0.0019	0.738	0.8335	0.0005
RTC-119	1.5*^{a}	0.6^{b}	0.6^{b}	0.003^{c}	0.20	0.1	0.3	0.4	0.7	0.08							
RTC-361	0.3	0.7	0.5	0.7	0.06	0.9	0.3	0.7	0.4	0.10							
RTC-400	0.4	0.3	0.5	0.7	0.07	1.1	1.0	1.1	1.0	0.09							

Lactic acid concentration (g/100g DM) at day 5

	Water-limited (75%)					Well-watered (100%)					Effect (*P*-value)						
**Genotype**	CTL[1]	HI[2]	MOL[3]	MOL+HI[4]	SEM	CTL	HI	MOL	MOL+HI	SEM	Irr[5]	Gen[6]	Add[7]	IrrxGen[8]	IrrxAdd[9]	GenxAdd[10]	IrrxGenxAdd[11]
RTC-2	1.2	0.7	2.7	0.8	0.42	0.3	0.8	1.0	0.7	0.14	<.0001	0.0035	0.0058	0.0023	0.0817	0.6464	0.511
RTC-119	1.0	0.4	0.7	0.4	0.10	0.3	0.5	0.9	1.1	0.15							
RTC-361	2.2	1.3	5.2*	2.3	0.82	0.4	0.2	0.4	0.2	0.05							
RTC-400	3.2	1.6	3.0	1.4	0.37	1.1	1.6	2.1	0.9	0.32							

The effect of irrigation, genotype and additives on WSC of tef silage at day 0 and 5 days after aerobic exposure

Water Soluble Carbohydrates (g/100g DM) at day 0

	Water-limited (75%)					Well-watered (100%)					Effect (*P*-value)						
**Genotype**	CTL[1]	HI[2]	MOL[3]	MOL+HI[4]	SEM	CTL	HI	MOL	MOL+HI	SEM	Irr[5]	Gen[6]	Add[7]	IrrxGen[8]	IrrxAdd[9]	GenxAdd[10]	IrrxGenxAdd[11]
RTC-2	0.13	0.02	0.06	0.02	0.03	0.04	0.02	0.07	0.11	0.02	0.0453	<.0001	<.0001	0.0009	0.0001	<.0001	0.0348
RTC-119	0.08	0.06	0.03	0.03	0.01	0.02	0.03	0.06	0.05	0.01							
RTC-361	0.11	0.02	0.10	0.04	0.02	0.10	0.12	0.08	0.05	0.01							
RTC-400	0.56*^{a}	0.03^{c}	0.17^{bc}	0.04^{c}	0.08	0.25^{a}	0.03^{b}	0.05^{b}	0.04^{b}	0.03							

Water Soluble Carbohydrates (g/100g DM) at day 5

	Water-limited (75%)					Well-watered (100%)					Effect (*P*-value)						
**Genotype**	CTL[1]	HI[2]	MOL[3]	MOL+HI[4]	SEM	CTL	HI	MOL	MOL+HI	SEM	Irr[5]	Gen[6]	Add[7]	IrrxGen[8]	IrrxAdd[9]	GenxAdd[10]	IrrxGenxAdd[11]
RTC-2	0.5*^{a}	0.1^{b}	0.2^{b}	0.03^{b}	0.07	0.1	0.01	0.1	0.1	0.01	<.0001	<.0001	<.0001	0.0017	<.0001	<.0001	0.0043
RTC-119	0.1	0.01	0.02	0.04	0.01	0.1	0.1	0.1	0.1	0.01							
RTC-361	0.3	0.1	0.2	0.3	0.04	0.1	0.2	0.1	0.1	0.02							
RTC-400	0.5*^{a}	0.1^{b}	0.2^{b}	0.1^{b}	0.06	0.3^{a}	0.1^{b}	0.1^{b}	0.1^{b}	0.02							

The effect of irrigation, genotype and additives on ammonia nitrogen of tef silage at day 0 and 5 days after aerobic exposure

Ammonia nitrogen concentration (mg/dL) at day 0

	Water-limited (75%)					Well-watered (100%)					Effect (*P*-value)						

Genotype	CTL[1]	HI[2]	MOL[3]	MOL+HI[4]	SEM	CTL	HI	MOL	MOL+HI	SEM	Irr[5]	Gen[6]	Add[7]	IrrxGen[8]	IrrxAdd[9]	GenxAdd[10]	IrrxGenxAdd[11]
RTC-2	4.1*	4.5	5.0	0.7	0.81	10.5[a]	8.9[ab]	9.4[ab]	1.6[c]	1.28	0.0029	0.0317	0.0608	0.0334	0.0174	0.0082	0.4183
RTC-119	4.2	3.5	2.3	3.8	0.40	5.4	6.2	3.5	1.2	0.76							
RTC-361	3.2	3.0	2.5	4.2	0.40	2.1	6.1	7.3	4.7	0.65							
RTC-400	2.4	3.0	3.2	6.3	0.63	2.9	2.5	4.2	3.5	0.38							

Ammonia nitrogen concentration (mg/dL) at day 5

	Water-limited (75%)					Well-watered (100%)					Effect (*P-v*alue)						
Genotype	CTL[1]	HI[2]	MOL[3]	MOL+HI[4]	SEM	CTL	HI	MOL	MOL+HI	SEM	Irr[5]	Gen[6]	Add[7]	IrrxGen[8]	IrrxAdd[9]	GenxAdd[10]	IrrxGenxAdd[11]
RTC-2	3.2*	4.1	3.2	0.9	0.58	9.6	9.4	9.0	4.7	0.77	<.0001	0.0245	0.6911	0.0253	0.7688	0.3612	0.4910
RTC-119	4.2	3.0	1.6	1.8	0.41	3.1	4.4	3.7	5.2	0.79							
RTC-361	1.2	1.4	2.5	2.6	0.34	6.9	5.7	7.7	3.9	0.61							
RTC-400	0.4	3.5	3.0	3.1	0.63	4.6	3.8	3.2	4.9	0.40							

The effect of irrigation, genotype and additives on OM content of tef silage at day 0 and day 5 of aerobic exposure

Organic Matter content (%) at day 0

	Water-limited (75%)					Well-watered (100%)					Effect (*P-v*alue)						
Genotype	CTL[1]	HI[2]	MOL[3]	MOL+HI[4]	SEM	CTL	HI	MOL	MOL+HI	SEM	Irr[5]	Gen[6]	Add[7]	IrrxGen[8]	IrrxAdd[9]	GenxAdd[10]	IrrxGenxAdd[11]
RTC-2	90.7*	90.4	90.0	90.0	0.12	88.4	88.1	88.0	88.7	0.16	<.0001	<.0001	0.0029	<.0001	0.7910	0.1645	0.1082
RTC-119	89.8	89.2	89.0	90.2	0.20	90.3	90.4	89.7	90.3	0.19							
RTC-361	91.5*	90.6	90.5	90.1	0.25	88.8	89.3	89.3	88.9	0.14							
RTC-400	92.0*	91.8*	91.4	91.3	0.14	89.9[a]	89.2[a]	87.9[b]	88.1[ab]	0.26							

Organic Matter content (%) at day 5

	Water-limited (75%)					Well-watered (100%)					Effect (*P-v*alue)						
Genotype	CTL[1]	HI[2]	MOL[3]	MOL+HI[4]	SEM	CTL	HI	MOL	MOL+HI	SEM	Irr[5]	Gen[6]	Add[7]	IrrxGen[8]	IrrxAdd[9]	GenxAdd[10]	IrrxGenxAdd[11]
RTC-2	92.0*	91.6*	91.0*	91.6	0.18	89.0	88.9	88.3	89.3	0.22	<.0001	<.0001	0.0672	<.0001	0.5623	0.4956	0.9935
RTC-119	90.0	89.2	89.6	89.4	0.17	90.3	90.2	90.8	89.6	0.21							
RTC-361	91.1	90.7	90.8	90.8	0.12	89.1	88.9	88.9	88.9	0.14							
RTC-400	92.8*	92.0	91.8	91.8	0.19	90.4	90.3	89.9	89.7	0.18							

The effect of irrigation, genotype and additives on Crude Protein content on tef silage at day 0 and 5 days after aerobic exposure

Crude Protein Content (%) at Day 0

	Water-limited (75%)					Well-watered (100%)					Effect (*P-v*alue)						
Genotype	CTL[1]	HI[2]	MOL[3]	MOL+HI[4]	SEM	CTL	HI	MOL	MOL+HI	SEM	Irr[5]	Gen[6]	Add[7]	IrrxGen[8]	IrrxAdd[9]	GenxAdd[10]	IrrxGenxAdd[11]

RTC-2	15.8	15.8	15.7	16.9	0.24	13.9	13.3	13.8	14.2	0.16	<.0001	<.0001	0.0315	<.0001	0.6669	0.4151	0.2660
RTC-119	12.2	12.8	11.4	12.3	0.23	14.0	13.3	12.3	14.0	0.30							
RTC-361	14.9	14.5	15.5	15.0	0.25	14.6	14.8	13.3	14.5	0.20							
RTC-400	14.8*	15.0	15.0	16.0	0.24	12.0	12.6	12.7	12.7	0.24							

Crude Protein Content (%) at Day 5

	Water-limited (75%)					Well-watered (100%)					Effect (*P-v*alue)						
Genotype	CTL[1]	HI[2]	MOL[3]	MOL+HI[4]	SEM	CTL	HI	MOL	MOL+HI	SEM	Irr[5]	Gen[6]	Add[7]	IrrxGen[8]	IrrxAdd[9]	GenxAdd[10]	IrrxGenxAdd[11]
RTC-2	14.9	16.0	16.2	15.6	0.30	14.8	14.7	15.7	16.2	0.26	0.0190	<.0001	0.0181	0.0004	0.6678	0.9110	0.6697
RTC-119	12.2	12.7	11.9	13.0	0.22	12.9	12.9	13.8	13.1	0.13							
RTC-361	13.5	14.6	14.3	13.7	0.30	13.1	13.9	13.7	13.7	0.16							
RTC-400	13.8	15.3	15.9	15.2	0.32	12.9	13.6	13.1	13.2	0.30							

The effect of irrigation, genotype and additives on NDF content of tef silage at day 0 and 5 days after aerobic exposure

NDF Content (%) at day 0

	Water-limited (75%)					Well-watered (100%)					Effect (*P-v*alue)						
Genotype	CTL[1]	HI[2]	MOL[3]	MOL+HI[4]	SEM	CTL	HI	MOL	MOL+HI	SEM	Irr[5]	Gen[6]	Add[7]	IrrxGen[8]	IrrxAdd[9]	GenxAdd[10]	IrrxGenxAdd[11]
RTC-2	63.1	66.6	64.9*	65.2	0.99	70.0	70.0	74.7	73.8	0.92	<.0001	<.0001	0.1784	<.0001	0.2621	0.0008	0.0069
RTC-119	62.7	64.2	68.2	53.6	2.14	60.8	64.9	63.8	62.3	0.94							
RTC-361	61.9	61.4	58.1	64.0	0.97	64.0	61.7	62.8	61.2	0.64							
RTC-400	62.4	64.7	62.5	63.8	0.59	68.9	69.5	69.3	70.4	0.38							

NDF Content (%) at day 5

	Water-limited (75%)					Well-watered (100%)					Effect (*P-v*alue)						
Genotype	CTL[1]	HI[2]	MOL[3]	MOL+HI[4]	SEM	CTL	HI	MOL	MOL+HI	SEM	Irr[5]	Gen[6]	Add[7]	IrrxGen[8]	IrrxAdd[9]	GenxAdd[10]	IrrxGenxAdd[11]
RTC-2	64.2	66.4	68.0	64.6	0.90	70.9	71.9	74.1	70.9	0.63	<.0001	<.0001	0.1378	<.0001	0.3877	0.2874	0.7473
RTC-119	70.1	70.0	69.5	70.2	0.45	65.4	65.5	67.7	67.9	0.54							
RTC-361	62.2	67.6	63.7	66.7	1.13	64.2	66.3	66.3	64.2	0.52							
RTC-400	64.7*	67.3	65.4	65.4	0.64	72.5	71.2	70.7	72.1	0.57							

The effect of irrigation, genotype and additives on ADF content of tef silage at day 0 and 5 days after aerobic exposure

ADF Content (%) at day 0

	Water-limited (75%)					Well-watered (100%)					Effect (*P-v*alue)						
Genotype	CTL[1]	HI[2]	MOL[3]	MOL+HI[4]	SEM	CTL	HI	MOL	MOL+HI	SEM	Irr[5]	Gen[6]	Add[7]	IrrxGen[8]	IrrxAdd[9]	GenxAdd[10]	IrrxGenxAdd[11]
RTC-2	34.5	36.9	35.8*	36.7	0.88	39.7[bc]	42.0[bc]	54.6[a]	44.0[b]	2.32	<.0001	<.0001	0.7011	<.0001	0.1912	0.0037	0.0008

RTC-119	35.8a^{b}	34.9^{ab}	38.9^{a}	28.0^{b}	1.54	30.9	33.1	32.9	34.5	0.69
RTC-361	34.5	33.0	30.9	35.7	0.77	34.0	31.5	33.0	31.6	0.55
RTC-400	34.6	33.8	33.0	34.6	0.52	38.9	38.8	38.3	38.8	0.32

ADF Content (%) at day 5

	Water-limited (75%)					Well-watered (100%)					Effect (*P-v*alue)						
Genotype	CTL[1]	HI[2]	MOL[3]	MOL+HI[4]	SEM	CTL	HI	MOL	MOL+HI	SEM	Irr[5]	Gen[6]	Add[7]	IrrxGen[8]	IrrxAdd[9]	GenxAdd[10]	IrrxGenxAdd[11]
RTC-2	34.9*	36.2	37.6*	35.0	0.64	43.9	43.6	46.0	42.6	0.64	<.0001	0.0021	0.1519	<.0001	0.1675	0.2588	0.6960
RTC-119	39.4	39.2	40.1	40.5	0.36	38.5	39.7	42.2	38.0	0.67							
RTC-361	33.7	38.2	35.1	37.2	0.98	39.2	40.1	39.9	38.0	0.49							
RTC-400	35.2*	36.2	35.5	34.6	0.53	44.5	41.8	41.2	41.6	0.57							

The effect of irrigation, genotype and additives on Hemicellulose content of tef silage at day 0 and 5 days after aerobic exposure

Hemicellulose Content (%) at day 0

	Water-limited (75%)					Well-watered (100%)					Effect (*P-v*alue)						
Genotype	CTL[1]	HI[2]	MOL[3]	MOL+HI[4]	SEM	CTL	HI	MOL	MOL+HI	SEM	Irr[5]	Gen[6]	Add[7]	IrrxGen[8]	IrrxAdd[9]	GenxAdd[10]	IrrxGenxAdd[11]
RTC-2	25.1	26.9	25.5	25.4	0.29	27.5	28.3	28.2	28.3	0.34	<.0001	0.0021	0.0093	0.0503	0.1126	0.0018	0.7292
RTC-119	24.4^{ab}	24.0*^{ab}	27.9^{a}	18.9^{bc}	1.26	25.0^{a}	15.9^{b}	23.1^{a}	23.8^{a}	1.50							
RTC-361	26.4	24.4	24.4	26.0	0.69	27.0	24.5	26.4	24.5	0.54							
RTC-400	24.9	25.8	24.9	25.3	0.34	28.6	27.8	27.6	27.6	0.38							

Hemicellulose Content (%) at day 5

	Water-limited (75%)					Well-watered (100%)					Effect (*P-v*alue)						
Genotype	CTL[1]	HI[2]	MOL[3]	MOL+HI[4]	SEM	CTL	HI	MOL	MOL+HI	SEM	Irr[5]	Gen[6]	Add[7]	IrrxGen[8]	IrrxAdd[9]	GenxAdd[10]	IrrxGenxAdd[11]
RTC-2	28.8	29.3	29.4	29.0	0.22	28.5	33.0	32.3	31.0	0.93	<.0001	<.0001	<.0001	<.0001	0.001	0.004	0.0127
RTC-119	30.6	32.3	33.3	30.7	0.62	29.4	31.6	28.8	28.1	0.80							
RTC-361	28.8	24.5	27.0	26.2	0.90	27.6	29.7	28.5	27.5	0.54							
RTC-400	29.1	28.8	26.0	26.8	0.57	33.0	30.4	30.1	31.5	0.55							

The effect of irrigation, genotype and additives on Cellulose content of tef silage at day 0 and 5 days after aerobic exposure

Cellulose Content (%) at day 0

	Water-limited (75%)					Well-watered (100%)					Effect (*P-v*alue)						
Genotype	CTL[1]	HI[2]	MOL[3]	MOL+HI[4]	SEM	CTL	HI	MOL	MOL+HI	SEM	Irr[5]	Gen[6]	Add[7]	IrrxGen[8]	IrrxAdd[9]	GenxAdd[10]	IrrxGenxAdd[11]
RTC-2	28.6	29.7	29.1	28.5	0.23	30.2	28.0	29.2	29.8	0.36	0.5967	0.0006	0.321	0.0681	0.0489	0.0169	0.0311
RTC-119	26.9	29.3	29.3	25.6	0.80	29.9	31.8	31.0	27.8	0.63							

RTC-361	27.4	28.4	27.2	28.3	0.28	30.0	30.2	29.8	29.7	0.27							
RTC-400	27.9	30.9	29.4	29.2	0.44	30.0	30.7	31.0	31.6	0.27							

Cellulose Content (%) at day 5

	Water-limited (75%)					Well-watered (100%)					Effect (*P-v*alue)						
**Genotype**	**CTL**[1]	**HI**[2]	**MOL**[3]	**MOL+HI**[4]	**SEM**	**CTL**	**HI**	**MOL**	**MOL+HI**	**SEM**	**Irr**[5]	**Gen**[6]	**Add**[7]	**IrrxGen**[8]	**IrrxAdd**[9]	**GenxAdd**[10]	**IrrxGenxAdd**[11]
RTC-2	29.3	30.2	30.4	29.6	0.37	27.0	28.3	28.10	28.3	0.25	0.0119	0.0001	0.497	0.0013	0.4613	0.227	0.4103
RTC-119	30.7*	30.7*	29.4*	29.7	0.27	26.9^{a}	25.8^{a}	25.52^{a}	29.9^{b}	0.68							
RTC-361	28.6*	29.5*	28.6*	29.6*	0.20	25.0	26.3	26.31	26.2	0.20							
RTC-400	29.5	31.1	29.9	30.7	0.26	28.0^{bc}	29.3^{ab}	29.48^{ab}	30.6^{a}	0.28							

The effect of irrigation, genotype and additives on ADL content of tef silage at day 0 and 5 days after aerobic exposure

ADL Content (%) at day 0

	Water-limited (75%)					Well-watered (100%)					Effect (*P-v*alue)						
**Genotype**	**CTL**[1]	**HI**[2]	**MOL**[3]	**MOL+HI**[4]	**SEM**	**CTL**	**HI**	**MOL**	**MOL+HI**	**SEM**	**Irr**[5]	**Gen**[6]	**Add**[7]	**IrrxGen**[8]	**IrrxAdd**[9]	**GenxAdd**[10]	**IrrxGenxAdd**[11]
RTC-2	9.4^{ab}	6.1^{*a}	10.2^{b}	11.0^{*b}	0.75	11.0^{c}	12.1^{bc}	16.4^{a}	11.7^{bc}	0.79	0.0013	<.0001	0.0193	<.0001	<.0001	<.0001	<.0001
RTC-119	10.6*	10.2*	10.0	8.4	0.36	5.4^{a}	14.9^{bc}	6.6^{c}	9.9^{c}	1.40							
RTC-361	7.9	8.0	6.2	9.3	0.47	6.7	6.7	6.2	6.6	0.17							
RTC-400	9.5	7.7	6.5	9.0	0.46	9.3	9.8	8.1	9.9	0.30							

ADL Content (%) at day 5

	Water-limited (75%)					Well-watered (100%)					Effect (*P-v*alue)						
**Genotype**	**CTL**[1]	**HI**[2]	**MOL**[3]	**MOL+HI**[4]	**SEM**	**CTL**	**HI**	**MOL**	**MOL+HI**	**SEM**	**Irr**[5]	**Gen**[6]	**Add**[7]	**IrrxGen**[8]	**IrrxAdd**[9]	**GenxAdd**[10]	**IrrxGenxAdd**[11]
RTC-2	5.6*	6.6	7.7	5.9	0.54	14.3	9.8	13.1	11.0	1.15	<.0001	0.3104	0.4615	0.0462	0.0168	0.2229	0.1304
RTC-119	8.0	6.4	6.4	9.0	0.58	8.9	7.7	12.6	9.0	0.90							
RTC-361	4.6	13.1	7.6	10.6	1.24	11.4	9.9	11.1	10.1	0.45							
RTC-400	5.9	7.1	9.2	7.4	0.55	10.7	10.7	10.5	9.3	0.54							

The effect of irrigation, genotype and additives on % DM loss of tef silage at day 0 and 5 days after aerobic exposure

DM Loss (%) at day 0

	Water-limited (75%)					Well-watered (100%)					Effect (*P-v*alue)						
**Genotype**	**CTL**[1]	**HI**[2]	**MOL**[3]	**MOL+HI**[4]	**SEM**	**CTL**	**HI**	**MOL**	**MOL+HI**	**SEM**	**Irr**[5]	**Gen**[6]	**Add**[7]	**IrrxGen**[8]	**IrrxAdd**[9]	**GenxAdd**[10]	**IrrxGenxAdd**[11]
RTC-2	1.4	1.9	1.7	1.7*	0.07	2.0	2.1	2.0	1.8	0.06	0.046	0.346	0.0222	<.0001	<.0001	0.796	0.7716
RTC-119	1.8	2.2	1.9	2.1	0.08	1.6^{ab}	1.6^{ab}	1.6^{ab}	1.1^{a}	0.08							
RTC-361	1.5*	1.8	1.6	1.7	0.05	2.3	2.2	2.0	2.0	0.05							

RTC-400	1.3*	2.1	1.7	1.9	0.11	2.2	2.0	1.8	2.0	0.08						
**DM Loss (%) at day 5**																
	**Water-limited (75%)**					**Well-watered (100%)**					**Effect (*P*-*v*alue)**					
**Genotype**	**CTL[1]**	**HI[2]**	**MOL[3]**	**MOL+HI[4]**	**SEM**	**CTL**	**HI**	**MOL**	**MOL+HI**	**SEM**	**Irr[5]**	**Gen[6]**	**Add[7]**	**IrrxGen[8]**	**IrrxAdd[9]**	**GenxAdd[10]**	**IrrxGenxAdd[11]**
RTC-2	3.2	3.3	3.5	3.3	0.06	2.9	3.1	3.0	3.3	0.07	0.0296	0.0007	0.7096	<.0001	0.3728	0.6214	0.1562
RTC-119	3.3	3.5	3.4	3.7	0.08	3.4	3.1	3.3	2.7	0.16							
RTC-361	2.8*	3.3	3.1	3.4	0.10	4.6	4.3	3.9	4.0	0.13							
RTC-400	3.5	3.3	2.8	3.2	0.11	3.5	3.3	3.3	3.6	0.09							
**Moulds and Yeast did not grow on tef silage.**																	
**The carbondioxide emitted form tef silage did not reach the minimal detection levels.**																	

*Significant differences between irrigation regimes within the same treatment at P < 0.05 after Tukey HSD test, Least Square Means not connected by the same letters between treatments within the same irrigation regime are statistically different at P < 0.05 after Tukey HSD test, SEM: Standard error of the mean, CTL[1] – Control, HI[2] – Heterofermentative Inoculum, MOL[3] - Molasses, MOL+HI[4] – Molasses+ Heterofermentative Inoculum,, Irr – Irrigation[5], Gen[6]– Genotype, Add[7] – Additive, IrrxGen[8] - Irrigation genotype interaction, IrrxAdd[9] - Irrigation additive interaction and GenxAdd[10] -Genotype additive interaction and IrrxGenxAdd[11] – Irrigation, genotype and additive interaction.
